# Venom Diversity and Evolution in the Most Divergent Cone Snail Genus *Profundiconus*

**DOI:** 10.3390/toxins11110623

**Published:** 2019-10-28

**Authors:** Giulia Fassio, Maria Vittoria Modica, Lou Mary, Paul Zaharias, Alexander E. Fedosov, Juliette Gorson, Yuri I. Kantor, Mandё Holford, Nicolas Puillandre

**Affiliations:** 1 Department of Biology and Biotechnologies “Charles Darwin”, Sapienza University of Rome, 00185 Rome, Italy; 2 Department of Biology and Evolution of Marine Organisms, Stazione Zoologica Anton Dohrn, Villa Comunale, 80121 Naples, Italy; mariavittoria.modica@szn.it; 3 University of Montpellier, 34095 Montpellier, France; 4 Institut Systématique Evolution Biodiversité (ISYEB), Muséum National d’Histoire Naturelle, CNRS, Sorbonne Université, EPHE, Université des Antillles, 57 rue Cuvier, CP 26, 75005 Paris, France; loumaryupmc@gmail.com (L.M.); paul.zaharias@mnhn.fr (P.Z.); nicolaspuillandre@gmail.com (N.P.); 5 A.N. Severtzov Institute of Ecology and Evolution, Russian Academy of Sciences, Leninski prospect 33, 119071 Moscow, Russian; fedosovalexander@gmail.com (A.E.F.); kantor.yuri1956@gmail.com (Y.I.K.); 6 Department of Chemistry, Hunter College, CUNY, New York, NY 10065, USA; jmgorson@gmail.com; 7 Department of Invertebrate Zoology, The American Museum of Natural History, New York, NY 10024, USA; 8 Biology, Biochemistry, and Chemistry programs of The CUNY Graduate Center, New York, NY 10016, USA

**Keywords:** Conidae, conotoxins, turripeptides, transcriptome, venom gland

## Abstract

*Profundiconus* is the most divergent cone snail genus and its unique phylogenetic position, sister to the rest of the family Conidae, makes it a key taxon for examining venom evolution and diversity. Venom gland and foot transcriptomes of *Profundiconus* cf. *vaubani* and *Profundiconus* *neocaledonicus* were de novo assembled, annotated, and analyzed for differential expression. One hundred and thirty-seven venom components were identified from *P.* cf. *vaubani* and 82 from *P. neocaledonicus*, with only four shared by both species. The majority of the transcript diversity was composed of putative peptides, including conotoxins, profunditoxins, turripeptides, insulin, and prohormone-4. However, there were also a significant percentage of other putative venom components such as chymotrypsin and L-rhamnose-binding lectin. The large majority of conotoxins appeared to be from new gene superfamilies, three of which are highly different from previously reported venom peptide toxins. Their low conotoxin diversity and the type of insulin found suggested that these species, for which no ecological information are available, have a worm or molluscan diet associated with a narrow dietary breadth. Our results indicate that *Profundiconus* venom is highly distinct from that of other cone snails, and therefore important for examining venom evolution in the Conidae family.

## 1. Introduction

As stated by the “father” of toxicology, “*Omnia venenum sunt: nec sine veneno quicquam existit. Dosis sola facit, ut venenum non fit.*”: Everything can be a venom, only the dose makes the difference between a poisoning and a non-poisoning substance [1]. This intuition deftly summarized what happened independently several times across the animal kingdom, where gene families, encoding for proteins normally involved in key regulatory processes, were recruited for venom production [2]. Many of such gene families underwent positive selection and rapid expansion during the evolutionary history of venomous animal, showing the importance of defensive and predatory venoms in the prey-predator arms race [3]. Typically, venom is a complex mixture of different components generally referred to as ‘toxins’, which directly interfere with the physiology of the prey acting often as neurotoxins or enzymes impairing haemostasis [2,3]. However, they can be also listed as venom components those enzymes involved in post-translational modifications, folding processes, and in enhancing venom activity by easing toxins spreading [2,3].

Current studies of venomous animals allow for better understanding of convergent recruitment of venom component in distantly related taxa, prey–predator coevolution, and represent a starting point for the identification of new bioactive compounds of pharmaceutical interest. Among taxa that have developed a venomous function, the globally distributed gastropod superfamily Conoidea is a highly diversified group of carnivorous species including the family Conidae J. Fleming, 1822 [4,5]. Based on the latest published molecular phylogeny of the Conidae [6] six genera, comprising ~850 species, can be recognized: *Profundiconus* Kuroda, 1956, *Californiconus* J. K. Tucker & Tenorio, 2009, *Lilliconus* G. Raybaudi Massilia, 1994, *Pygmaeconus* Puillandre & Tenorio, 2017, *Conasprella* Thiele, 1929, and *Conus* Linnaeus, 1758. The Conidae venom arsenal, combined with sophisticated envenomation strategies, has allowed these slow-moving snail species to prey on worms, other molluscs, and fish [7]. Of the six Conidae genera, the complex venom cocktails of the genus *Conus* Linnaeus, 1758 (Conidae) have been intensively studied and contain mainly conotoxins that target ion channels, neurotransmitter transporters, and receptors of the nervous system of the prey [7]. A conotoxin precursor is typically composed of a conserved signal region, a pro-region that may be present or lacking, a highly variable mature peptide in most cases rich in cysteine residues, and a short terminal region [8]. The conotoxins are classified into gene superfamilies based on the percentage sequence identity (PID) of the signal region, and into cysteine patterns based on the number and distribution of cysteine residues in the mature region [8,9]. Data produced to date show that the conotoxin mixture produced by the venom gland varies not only at the inter- and intra-specific level [10,11], but also within a single individual over time [12], and among different regions of the venom gland [13]. In particular, the distal and the proximal segments of the venom gland, with respect to the venom bulb, produce different kinds of venom, in response to elicitation of a defensive or a predatory behaviour, respectively [13]. Despite the prevalence of conotoxins, other bioactive compounds have been found in conid venom, including insulins, c-type lectins, disulfide isomerase, and astacins [14,15,16,17,18,19,20]. 

Recent phylogenetic reconstructions, based on exomes and mitogenomes, confidently identified *Profundiconus* as the sister-lineage to all the other Conidae, dating its divergence from the other genera around 56–62 Mya [21,22]. As suggested by its name, the 28 extant species ascribed to this genus live in, but are not all restricted to, very deep waters, mainly in the Indo-Pacific [23]. The phylogenetic position of *Profundiconus*, as sister to all other cone-snails, makes it a key taxon for understanding conid venom evolution enabling to infer the venom composition of the ancestor of all present-day cone-snails.

Due to the lack of direct observations, *Profundiconus* ecology and hunting strategy could only be inferred from their radular morphology, which indicates a likely vermivory [24]. Additionally, the find of a small octopus beak in the stomach of a *Profundiconus smirnoides* Tenorio, 2015 specimen suggested the ability, of at least this species, to prey on cephalopods [24,25].

This study describes, for the first time, the venom composition of *Profundiconus*. The venom gland (VG) and foot (F) transcriptomes of *Profundiconus* cf. *vaubani* (Röckel & Moolenbeek, 1995) and *Profundiconus neocaledonicus* Tenorio & Castelin, 2016 were analyzed and novel gene superfamilies, as well as other venom components involved in toxicity, toxin processing, or in enhancing venom activity, are reported here. The novelty of gene superfamilies found, the presence of putative turripeptides, and the high abundance of other venom proteins suggest that *Profundiconus* venom gland transcriptomes are significantly different from those of other conids.

## 2. Results

### 2.1. Analysis of Profundiconus Transcriptomes

The transcriptome sequencing of three venom glands (from *Pvau1, Pvau2*, and *Pneo*) and two foot samples (from *Pvau2* and *Pneo*) provided a total of 179,582,083 paired raw reads, (M = 35,916,417; SD = 3,439,048). After assembling and filtering, the dataset was composed of 1,049,747 contigs (M = 209,949; SD = 114,381) (Table S1). VG contigs were translated into 11,070,979 ORFs, including 191,207 with a signal region. After filtering, by similarity with other venom components and by subcellular localization, and removing duplicates, 223 unique putative venom components were retrieved, including those enriched in the VG, all considered new because having ≥1 amino acid (aa) of difference from previously published sequences. The number of venom components varied between specimens, with 51 found in *Pvau1*, 108 in *Pvau2*, and 86 in *Pneo.* Of those, 18 were shared between the two *P.* cf. *vaubani* specimens, while 3 between *Pvau2* and *Pneo*, and 1 between *Pvau1* and *Pneo*. At the species level, 137 unique venom components were retrieved from *P.* cf. *vaubani*, 82 from *P. neocaledonicus*, and 4 were shared by both.

OrthoVenn2 was used to compare *Pvau2* and *Pneo* venom gland and foot transcriptomes. The analysis identified a total of 55,827 putative orthologous clusters (Figure S1). Overall, the four transcriptomes appeared to be quite different with a low percentage of clusters shared by all them (18%). The *Pvau2* and *Pneo* foot samples were the most diversified, showing a higher number of total clusters compared with the respective venom glands (*Pneo*: F 164,539 vs. VG 60,736; *Pvau2*: F 118,640 vs. VG 79,727). The analysis also detected a higher similarity between similar tissue types than between species. Specifically, the two venom gland transcriptomes, with 29,485 shared clusters, appeared to be overall the most similar. The foot samples showed also a higher number of tissue specific clusters (F 9046 vs. VG 2908). Whereas, the foot and the venom gland of *Pvau2* showed a lower overlap (25,606), comparable to the overlap between *Pneo* F and VG (24,342). 

Clusters were grouped by gene ontology (GO) process and molecular function classes. We did not detect a marked difference in the most abundant GO classes present in foot and in venom glands samples. However, when considering the less abundant GO classes, venom glands were more diversified. In particular, in both tissues the four most abundant GO process classes (56–61% of the total) were annotated as involved in “biological”, “cellular”, and “metabolic processes”, and “biological regulation”. In addition, in the venom glands, we found 5% of the clusters involved in “RNA metabolic processes”, while this group was not detected in the foot samples. The situation was similar regarding GO molecular functions classes, with “ion binding”, “nucleic acid banding”, and “hydrolase activity” among the most abundant ones in both tissue (55–61% of the total). In the venom glands, clusters labelled as “transporters” (5%) and “extracellular region” (2%) were also detected.

### 2.2. Molecular Types and Putative Targets of Profundiconus Venom Components

**Figure 1:**
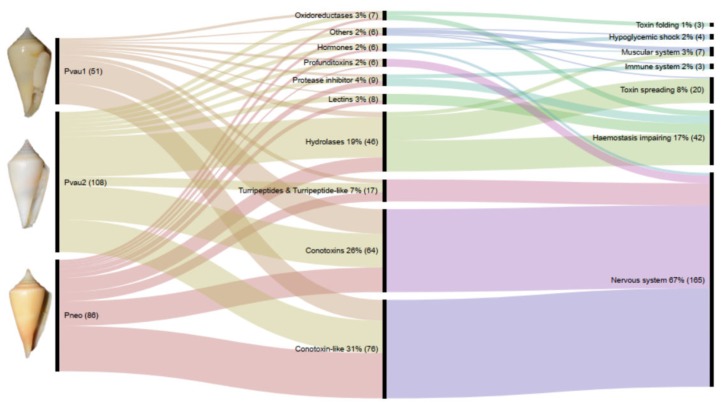
Comparison of *Profundiconus* venom peptides and their potential application. Shown is an alluvial diagram made with RAWGraphs [26] composed of three steps (vertical lines) summarizing types of peptides found in the venom gland (centre) of each specimen (left), and their potential physiological target or effects (right). Flows are proportional to number of peptides. Numbers represent percentages and absolute numbers (between parenthesis) of transcripts found in the relative category. On the left, voucher photos of dissected specimens: *P.* cf. *vaubani* 1 (MNHN-IM-2013-66002, 20.7 mm length), *P.* cf. *vaubani* 2 (MNHN-IM-2013-69344, 22 mm length), and *P. neocaledonicus* (MNHN-IM-2013-69343, 24.5 mm length); copyright MNHN.

**Figure 2:**
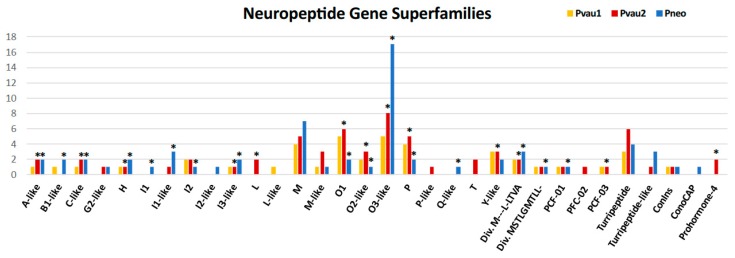
Number of putative neuropeptides found in the venom glands (VG) of *Profundiconus* specimens categorized by gene superfamilies. Asterisks indicate specimens and gene superfamilies in which overexpressed peptides were found.

**Figure 3:**
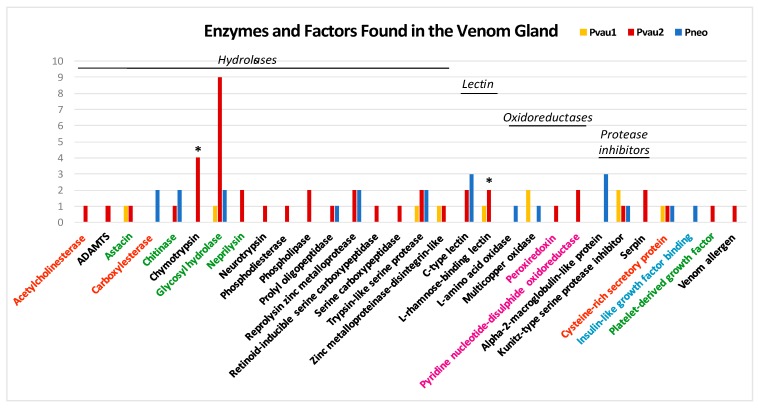
Number of non-neuropeptide venom components of *Profundiconus* specimens, categorized by family. Asterisks indicate the specimens and families in which overexpressed peptides were found. Category colours indicate putative compounds targets/effects: orange = muscular system; black = haemostasis impairing; green = easing toxin spreading; fuchsia = toxins folding; light blue = hypoglycaemic shock.

In all three *Profundiconus* VG transcriptomes, putative neuropeptides represented the most diversified group (59–80% of the total diversity, total *n* = 148), followed by the hydrolases (8–28%, *n* = 45), while oxidoreductases, protease inhibitors, and lectins were present only in small amounts (≤5%, *n* < 10) (Figure 1 and Figure S2). Unique isolated neuropeptides were identified as: Conotoxins (123), profunditoxins (for definition see Paragraph 5.4) (7), turripeptides (13), cono-insulin (2), cono-cardioactive peptide (1), and prohormone-4 (2) (Figure 2). Seventy-five unique peptides of other putative venom compounds were found, and more than half were exclusively present in *Pvau2* (*Pvau1* = 10, *Pvau2* = 44, and *Pneo* = 22) (Figure 3). A majority of the other putative venom components were represented by one or two types of peptides, with the exception of glycosyl hydrolases, which was particularly diversified in *Pvau2* (nine peptides).

Based on the potential molecular function of each transcript, 58–78% were putative toxins targeting the nervous system, 13–22% could affect haemostasis, while 2–12% could facilitate toxins spreading (Figure 1 and Figure S2). In all three *Profundiconus* VG specimens, a limited number of transcripts with other putative functions were also found, including toxins inducing a hypoglycaemic shock or impairing the muscular system. Only in *Pneo* and *Pvau2* were toxins targeting the immune system found, and only in *Pvau2* were venom components involved in toxin post-translational modifications and folding detected.

The differential expression analysis of the two specimens from which transcripts from both VG and F were available (*Pvau2* and *Pneo*) identified 41 transcripts overexpressed in the VG, all neuropeptides except two. In *Pneo*, 21 conotoxin precursors and one profunditoxin precursor were overexpressed in the VG, while in *Pvau2* 15 conotoxin precursors, one profunditoxin precursor, one prohormone-4 (neuropeptide), one chymotrypsin (peptidase S1, hydrolase), and one L-rhamnose-binding lectin appeared overexpressed (Figure 2 and Figure 3 and Figure S3 and Table S2).

### 2.3. Analysis of Putative Conotoxins and Profunditoxins Transcripts

Of the total 130 unique conotoxin and profunditoxin precursors, 37 were found in *Pvau1*, 53 in *Pvau2,* and 55 in *Pneo* (Figure S4). The three VG specimens did not share identical precursors, since only 15 precursors are shared among the two *P.* cf. *vaubani* specimens and one is shared by *Pvau1* and *Pneo*. As a result, 74 unique precursors for *P.* cf. *vaubani*, 54 for *P. neocaledonicus*, and 1 shared by both species were found (Figure S4).

**Figure 4:**
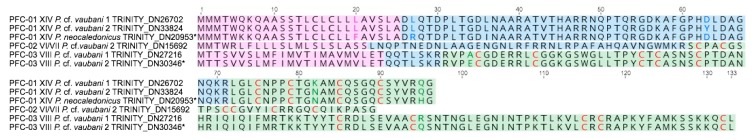
Putative *Profundiconus* new gene superfamilies PFC-01, PFC-02, and PFC-03 separately aligned. In the transcripts, signal regions are displayed in fuchsia colour, pro region in light blue, and putative mature peptide in green. Cysteine residues are coloured in red. Labels for each transcript indicate gene superfamily, cysteine framework, species, and sequence or transcript number. The asterisk (*) indicates *Profundiconus* sequences overexpressed in the venom gland.

Precursors were classified into 27 gene superfamilies, 17 of which appeared to be new (Table 1). Among the already known superfamilies (28,900 TPM from *Pneo* and *Pvau2*), conotoxin precursors were ascribed to the H, I1, I2, L, M, O1, P, and T superfamilies and to the two “divergent” superfamilies M---L-LTVA and MSTLGMTLL-, so called in the Conoserver database because they were first identified in the phylogenetically divergent species *Californiconus californicus* (Reeve, 1844) [27]. Among the remaining gene superfamilies (15,956 TPM from *Pneo* and *Pvau2*), 14 were similar to already described gene superfamilies (A-, B1-, C-, G2-, I1-, I2-, I3-, L-, M-, O2-, O3-, P-, Q-, and Y-like), while three were different from all conotoxins described so far, and therefore named profunditoxins (PFC-01, PFC-02, and PFC-03) (Figure 4). The three VG specimens did not show a great difference in number of identified gene superfamilies (*Pvau1* = 18, *Pvau2* = 22, and *Pneo* = 21) (Table 1 and Figure S4). More than half of the 15 gene superfamilies were shared by the three specimens, including the PFC-01 superfamily and the four most diversified ones (O3-like, M, O1, and P). Notably, eight gene superfamilies were unique to one of the studied specimens, namely: L-like in *Pvau1*; I1, I2-like and Q in *Pneo*; L, T, P-like and PFC-02 in *Pvau2*. Eighteen gene superfamilies were present in both species, while only six were found only in *P.* cf. *vaubani* (L, L-like, T, P-like, PFC-02, and PFC-03) and three in *P. neocaledonicus* (I1, I2-like, and Q). 

Among the profunditoxin gene superfamilies, PCF-01 showed a signal sequence of 25 aa, a pro-region of 46 aa, and a mature region of 25 aa. Three very similar sequences (2–6 divergent aa) were detected, one in each specimen, all sharing the XIV cysteine framework. The sequence identified in *Pneo* was overexpressed in the VG. The unique sequence of the PCF-02 superfamily was found in *Pvau2*, and it was composed of a 21 aa signal sequences, a 39 aa pro-region, and a 29 aa mature peptide showing the VI/VII cysteine pattern. Finally, two sequences with the VIII cysteine framework belonging to the PCF-03 superfamily were found, one in each *P.* cf. *vaubani* specimen. The sequence found in *Pvau2* was overexpressed in the VG. 

The 38 overexpressed conotoxin and profunditoxin precursors identified belonged to 19 gene superfamilies, 11 categorized as new, including PFC-01 and PFC-03 (Table 1). In *Pvau2*, 17 overexpressed precursors were found belonging to 13 gene superfamilies, while in *Pneo,* we retrieved 22 overexpressed precursors from 16 gene superfamilies. Seven precursors overexpressed in *Pvau2* were also found in *Pvau1*, for which it was not possible to compute contig differential expression. Nine gene superfamilies were overexpressed in both specimens, namely: A-like, C-like, H, I3-like, O1, O2-like, O3-like, P, and M---L-LTVA. Only three were found in *Pvau2* (L, Y-like, and PCF-02) and seven were found in *Pneo* (B1-like, I1, I1-like, I2, Q-like, PCF-01, and MSTLGMTLL-).

Conotoxin diversity values calculated by the Shannon’s Diversity Index were very similar between *Pvau1* (H’ = 2.68) and *Pneo* (H’ = 2.61), while slightly higher in *Pvau2* (H’ = 2.89). Instead, the Shannon’s Evenness Index was higher in the two *P.* cf. *vaubani* specimens (E_Pkan1_ = 0.81; E_Pkan2_ = 0.78) compared with the *P. neocaledonicus* one (E_Pter_ = 0.62). Combining results of the two *P.* cf. *vaubani* specimens, a species diversity index of 2.86 and an evenness of 0.69 were obtained.

A total of 19 different cysteine frameworks (including absence of cysteine) were found from the putative conotoxins, profunditoxins, and turripeptides, some of them present in more than one specimen (*Pvau1* = 12; *Pvau2* = 13; *Pneo* = 15). Ten of these frameworks were shared by all specimens: One shared by two, and six found in one specimen only (Table 1). The most abundant in all the three specimens were those with cysteine framework VI/VII (28–39%), followed by peptides without cysteine (15–19%). Thirteen conotoxin gene superfamilies had one or more cysteine frameworks that were not previously described for that superfamily. Five odd cysteine numbers (1 to 9) were also found in putative conotoxins, conotoxin-like, and turripeptides. Odd cysteine numbers have been reported from other cones species [15,27,28] and can be explained as belonging to homodimeric toxins [29] or originated from alternative RNA editing and splicing [30]. However, proteomic and experimental analyses will be needed to confirm that these odd numbered transcripts are toxins.

### 2.4. Analysis of Putative Turripeptide Trancripts

Nine unique putative turripeptides were identified (*Pvau1* = 3, *Pvau2* = 4, and *Pneo* = 3), presenting four cysteine frameworks (0, C, XVI, and IX), three of which shared by *Pvau2* and *Pneo* (Figure 2). Four unique turripeptides, present in *Pvau2* and *Pneo*, were similar to a previously identified turripeptide group characterized by a signal sequence similar to that of the B2 conotoxin gene superfamily, but lacking cysteine residues [31]. *Profundiconus* peptides belonging to this group lacked cysteine residues too and were similar to turripeptides found in *Gemmula speciosa* (Reeve, 1842) (D5KXH4), *Unedogemmula bisaya* (Olivera, 2004) (Ubs_01 and Ubs_15), and *Crassispira cerithina* (Anton, 1838) (Ccr_06 and Ccr_08). Two other peptides, found in all *Profundiconus* specimens, belonged to the turrid Pg gene superfamily, first identified in *G. speciosa* [32]. 

Four turripeptide-like compounds were also identified (*Pvau2* = 1 and *Pneo* = 3), all with the IX cysteine framework (Figure 2). Two of these sequences, found in *Pneo* only, were similar to a turripeptide found in *Iotyrris olangoensis* [33] (OL135-[34]) while the other two, found in *Pvau2* and *Pneo*, were similar to a turrid gene superfamily isolated by Olivera and colleagues [35]. This P-like superfamily is broadly expressed across turrids, but *Profundiconus* sequences appeared to be particularly similar to the sequence Pal9.2 from *Polystira albida* (G. Perry, 1811). In two turripeptide-like transcripts (TRINITY_DN29555 from *Pvau2* and TRINITY_DN20308 from *Pneo*) a kazal domain was found, indicating that they may also possess a serine protease inhibitor activity.

### 2.5. Analysis of Prohormone-4 Transcripts

**Figure 5:**
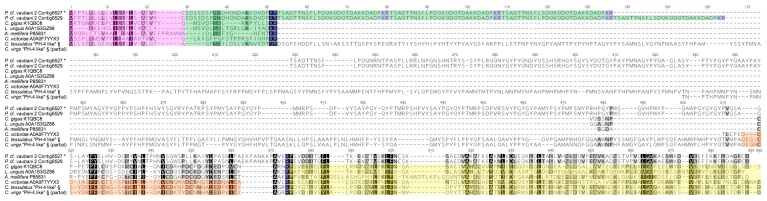
Alignment of *Profundiconus* Prohormone-4 precursors to known sequences from other taxa. In the transcripts, the signal regions are highlighted in fuchsia, putative mature peptides in green, cleavage sites in blue, Low-Density Lipoprotein (LDL) receptors in orange, and cysteine-free regions in yellow. Amino acids are coloured in greyscale based on Blosum62 similarity score (black 100%). The asterisk (*) indicates the *Profundiconus* sequence overexpressed in the venom gland. § indicates sequences from Robinson et al. [28] supplementary materials.

Two unique sequences of the neuropeptide prohormone-4 (PH-4) from *Pvau2* were isolated. One of these sequences (Contig6527) appeared overexpressed in the VG (Figure 5). Prohormone precursors are composed of a signal peptide, a short mature region delimited by a mono or dibasic cleavage site (KR), and a precursor-related peptide. In some cases, there can be multiple cleavage sites producing multiple mature peptides (polyprotein hormone precursor). *Profundiconus* PH-4 precursors were 539 (Contig6527) and 567 (Contig6529) aa long and structured as follows: i) A signal sequence; ii) a mature sequence, different from the others found so far, followed by a second type of mature peptide, repeated in tandem three times (in the overexpressed Contig6527) or four times (Contig6529), each delimited by predicted cleavage sites; iii) a long post region similar to the post region found in the “prohormone-4-like” *Conus* group; iv) two cysteines in the tail region of the sequence. This structure suggested that *Profundiconus* PH-4 is a polyprotein hormone precursor, producing two types of mature peptides, one of which in multiple copies.

PH-4 was first identified in the honeybee and linked to foraging predisposition (preference in collecting nectar or pollen) [36]. It has since been found in the brain of male eastern rock lobster [37] and in the venom gland of *Conus victoriae, Conus geographus, Conus tessulatus, Conus varius*, and *Conus virgo* [28], however its role in the envenomation process remains to be determined. *Conus* PH-4 has a mature sequence similar to the one retrieved in the honeybee and a precursor-related peptide containing an LDL (Low-Density Lipoprotein) receptor A and a cysteine free tail. Noteworthily, Robinson and colleagues (2017) found a second class of ‘‘prohormone-4-like” that is characterized by a longer precursor-related peptide (before the LDL receptor) and a different mature sequence. Of this second group, only one complete sequence was identified from *C. tessulatus*, but partial sequences were found in *Conus marmoreus*, *Conus victoriae*, *Conus bullatus*, *Conus geographus*, *Conus virgo*, and *Conus varians* [28].

### 2.6. Analysis of Insulin Transcripts

Two sequences of *Profundiconus* insulin were isolated: One in the two specimens of *P.* cf. *vaubani,* the other in *P. neocaledonicus* only (Figure 2). In molluscs, this well-known peptide hormone acts as a regulator of the glucose metabolism and as a neurotransmitter, and is derived from a precursor composed of a signal sequences and two chains (A and B) linked by a connecting peptide (C) [38]. In cone snails, two types of insulin have been found so far: A conventional signalling insulin, expressed in the nerve ring and probably involved in mollusc energy metabolism, and a derived insulin, expressed in the venom gland and involved in prey capture [20]. While the former is presumably present in all conids, with a conserved sequence, the latter was only detected in some species and shows higher variability [20]. Moreover, differences have been observed between venom insulin produced by fish-hunter *Conus* species compared with that of mollusc- and worm-hunter species. As a general trend, fish-hunter *Conus* venom insulin is similar to insulin found in fish, while mollusc- and worm-hunting *Conus* insulins are more similar to other mollusc signalling insulins [20,39].

The two *Profundiconus* predicted insulins were 129–130 aa length and had a sequence identity of 84% for the A chain and 74% for the B chain. The insulin chains of *Profundiconus* species showed different results when compared with those from other *Conus* species (Table 2 and Figure S5). Overall, *Profundiconus* insulin chains showed high similarity and identity with those from worm- or mollusc-hunting cones. While B chains from both *Profundiconus* species did not show a higher affinity to any one of these two groups, *P. neocaledonicus* insulin A chain was clearly more similar to molluscivorous *Conus (Cylinder) textile* Linnaeus, 1758. Alternatively, the A chain of the insulin from *P.* cf. *vaubani* showed slightly higher similarity to the A chain of three species of worm-hunting cones: *Conus (Lividoconus) floridulus* A. Adams & Reeve, 1848, *Conus (Virroconus) ebraeus* Linnaeus, 1758, and *Conus (Strategoconus) planorbis* Born, 1778. Notably, *Profundiconus* insulins also showed one additional cysteine residue on each chain, a feature present in venom insulins of worm- and mollusc-hunters, and also in signalling insulins from conids and other molluscs. These cysteines are supposed to form a third interchain disulfide bond, in addition to the two already existing between the chains A and B [39].

### 2.7. Analysis of Lectin Transcripts

**Figure 6:**
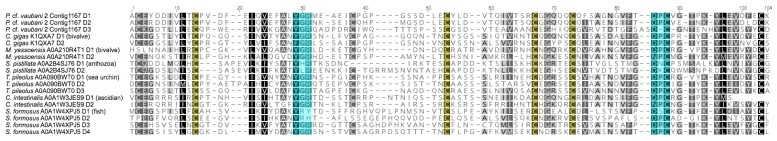
Alignment of *Profundiconus* L-rhamnose-binding lectin domain and other marine animals. Highlighted in light blue are the two conserved L-rhamnose-binding lectin domain motifs (YGR and DPC), responsible for ligand specificity. The four conserved cysteines are in yellow. Amino acids are coloured in greyscale based on Blosum62 similarity score (black 100%). In transcript sequence names, “DX” indicates the domain number.

Eight *Profundiconus* transcripts were identified as lectins. Five of the sequences found in *Pneo* and *Pvau2* were identified as C-type lectin, while three were L-rhamnose-binding lectin (RBL) from the two specimens of *P.* cf. *vaubani*. RBLs are composed of one to three tandem carbohydrate recognition domains and have been classified in five types according to the number of tandem domains. Each domain is characterized by two conserved motifs (YGR and DPC) responsible for ligand specificity, and four conserved disulfide bounds [40]. One complete type I RBL sequence (three tandem domains) was found in *Pvau2* (Contig1167) and overexpressed in the VG (Figure 6). The three domains are similar in sequences to the corresponding domain in *Crassostrea gigas* (Thunberg, 1793) (35–45% depending on the domain), but structurally homologous to those from the venomous sea urchin *Toxopneustes pileolus* (Lamarck, 1816) [41]. The two first domains have conserved motifs, while the third has a proline deletion in the DPC motif. Modifications of the first and second aa of this motif were found in anthozoan, bivalves, ascidian, and fish, however, this is the first reported finding of deletions.

RBLs have been mainly studied from fish eggs [42,43] and from globiferous pedicellariae of *T. pileolus* [41]. They were found also in colonial ascidians [44] and in bivalves, like the penguin wing oyster [45]. RBLs are involved in several processes including mitogenic, chemotactic, cytolytic, and apoptotic activities [41,46,47]. They also showed hemagglutination activity on rabbit erythrocytes, that can be inhibited by L-rhamnose and/or other saccharides [48]. 

### 2.8. Analysis of Chymotrypsin Transcripts

Three complete serine protease chymotrypsin-like sequences were found in *Pvau2*, one of which (TRINITY_DN34379) was overexpressed in the VG (Figure S6). Serine proteases are digestive enzymes characterized by a conserved catalytic triad (H/D/S) involved in the enzymatic activity. However, in some unconventional serine proteases, this triad is modified, like in the sedolisin proteases found in *Pseudomonas* sp., which have a different catalytic triad (S/D/E) and are active also at low pH conditions, e.g., hot springs or human lysosomes [49,50]. Among serine proteases, chymotrypsins are proteolytic enzymes involved in several intra- and extracellular activities. As venom components, they act on the coagulation cascade, on the fibrinolytic systems, and on platelets causing imbalance of the prey haemostatic system [51]. *Profundiconus* transcripts displayed sequence similarity with bivalves (30%) and snakes (25%) serine proteases, and structural similarity with chymotrypsins. However, the catalytic triad is the same as in *Pseudomonas* sp. sedolisin (S/D/E). 

## 3. Discussion

This study represents the first portrait of the venom composition of two *Profundiconus* species, *P.* cf. *vaubani* and *P. neocaledonicus*. The sister position of *Profundiconus* to the rest of the family Conidae [21,22] makes it an important piece to the conid venom puzzle for interpreting conotoxin diversity and evolution. Our findings suggest that the *Profundiconus* venom arsenal is significantly dissimilar to those of other *Conus*, in particular for the gene superfamilies found, the presence of putative turripeptides, and the high abundance of other venom proteins.

### 3.1. P. cf. Vaubani Displayed a High Diversity of Nonneuropeptide Venom Components

While all the VG transcriptomes appeared largely similar in terms of putative conotoxin and turripeptide diversity, *Pvau2* showed a definitively higher number of other venom components. Because prior conid literature has focused on neuropeptides, few works discussed the presence and diversity of other molecular types of venom components. Therefore, it is difficult to determine if the high percentages of non-neuropeptide sequences observed in *P. vaubani* can be considered ubiquitous among conids or peculiar to this species.

*P. cf. vaubani* venom appeared to be composed, in large part, of putative toxins potentially involved in haemostasis impairing, affecting prey muscular and immune system, and a smaller proportion of enzymes responsible for correct toxin folding or facilitating their spreading into prey tissues. For example, RBLs and chymotrypsins, which impact the coagulation cascade, were found exclusively in *P.* cf. *vaubani* specimens and enriched in *Pvau2* VG, suggesting potential use for impairing prey haemostasis. These two compounds are both found in other animal venoms, and were overexpressed in *P.* cf. *vaubani* VG, which suggests they may have an important role in the envenomation process. Of note is the *P.* cf. *vaubani* chymotrypsin, which is similar to chymotrypsins active at low pH, and could represent an adaptation to a specific hunting strategy or prey type. Another interesting protein overexpressed in the VG of *P.* cf. *vaubani* is PH-4. *P.* cf. *vaubani* PH-4 showed a different structure and mature peptide sequence from those found in other conids and in honeybees, and possessed two types of mature peptides, one of which in multiple copies. In honeybees, PH-4 was linked to foraging-related behaviour, however the lack of information concerning *Profundiconus* ecology and hunting strategy hampers the evaluation of PH-4, RBLs, and chymotrypsins and their potential effects on *P.* cf. *vaubani* prey.

### 3.2. Limited Conotoxin Diversity May Indicate a Narrow Worm or Molluscan Diet

Analysis of our transcriptome assemblies recovered different numbers of putative conotoxin and profunditoxin precursors: 75 for *P.* cf. *vaubani* and 55 for *P. neocaledonicus*. If the three specimens are considered independently, the number of putative conotoxin and profunditoxin precursors are: 37 (*Pvau1*), 53 (*Pvau2*), and 55 (*Pneo*). If only the conid species sequenced in conditions similar to the ones used in the present work (one to three VG samples, sequenced by Illumina HiSeq2000 platform) are taken into account, *P. neocaledonicus* emerges as the species showing the lowest number of conotoxin and contoxin-like precursors. *P.* cf. *vaubani* showed instead a higher number of conotoxin precursors, equal to the one in *Conus (Gastridium) geographus* Linnaeus, 1758 [52] and *C. (Virroconus) ebraeus* [53], from which, however, only one specimen was sequenced. Therefore, if we take into consideration only the result of one specimen for each *Profundiconus* species (*Pneo*, and the most numerous *Pvau2* with 53 conotoxins), *P. neocaledonicus* and *P.* cf. *vaubani* may be included among those conids with a less diversified neuropeptide arsenal.

Conversely, when the numbers of gene superfamilies produced in the same sequencing condition are considered, *P.* cf. *vaubani* and *P. neocaledonicus* show a high number of superfamilies (24 and 21, respectively) comparable to species with higher numbers of conotoxins, like *Conus (Virgiconus) virgo* Linnaeus, 1758 with 25 gene superfamilies and 113 conotoxins [53] and *C. (Gastridium) geographus* Linnaeus, 1758 with 21 gene superfamilies and 75 conotoxins [52]. The two *Profundiconus* species display a similar profile in terms of types and abundance of gene superfamilies. In fact, although with different percentages, the superfamilies O3-like, M, O1, and P appeared to be the most diversified in all the three *Profundiconus* specimens. The superfamilies M and O1 are among the most common and abundant conid gene superfamilies (e.g., [49,50]), so it is not unexpected to find them well represented in *Profundiconus*. The O3 superfamily, the most closely related to the newly described O3-like superfamily, is present in several species, but not abundant throughout conids. It was found in small numbers (up to seven different conotoxins) in 13 conid species with different feeding habits such as fish-, mollusc-, and worm-hunting [53,54]. Interestingly, this gene superfamily, along with the J and T ones, were overexpressed in the distal part of the VG of *C. (Gastridium) geographus*, accounting for ~50% of the total conotoxin reads found in this segment that is supposed to be the one producing predation venom, but for only ~5% in the proximal segments of the VG, the one producing defence-evoked venom [13,55]. The P superfamily was found in several fish-, mollusc-, and worm-hunting *Conus* species but always with low diversity (up to 14 different conotoxins), with the exception of *Conus (Turriconus) praecellens* A. Adams, 1855, in which this superfamily was the most abundant and diversified [52]. Remarkably, conotoxins belonging to the T gene superfamily, which are frequently common and diversified in the other conid species, are lacking in the venom gland transcriptome of the *Profundiconus* species analyzed here.

The overexpressed conotoxin and profunditoxin fraction, as calculated by differential expression analysis on TMP values, was quite similar in composition between *P.* cf. *vaubani* and *P. neocaledonicus*. In fact, 40% of the gene superfamilies found were overexpressed in both species, and overall, 50–70% of them included at least one transcript overexpressed in the VG. These results suggest that the venom cocktails of *P.* cf. *vaubani* and *P. neocaledonicus* may be not identical but for a large part similar, at least for what concern the conotoxin composition.

Comparing *Profundiconus* Shannon’s diversity and evenness indexes with those of other conid species sequenced in similar conditions, like *C. tribblei* (H’ = 3.30, E = 0.90) and *C. lenevati* (H’ = 3.30, E = 0.89) [56], confirmed what was already suggested by conotoxins abundance. *P.* cf. *vaubani* and *P. neocaledonicus* showed slightly lower conotoxin diversities compared with other species and a less homogeneous distribution of transcripts among gene superfamilies. However, the pipeline we used to identify putative toxins in *Profundiconus* tissues was largely based on similarity with conotoxins, potentially leaving highly divergent profunditoxins undetected, and leading to an underestimation of the real toxin diversity in *Profundiconus*. Pipelines that do not rely mainly on similarity with previously described toxins but more on, e.g., their structural properties, are required to detect the eventual new toxins of this divergent genus that may be different from what found in *Conus* up to now. This is particularly true when it is taken into account that the range of conotoxin precursors and gene superfamilies reported, the number of specimens used (1–20), and the types of sequencing platforms (five types) vary greatly among the conid VG transcriptomes produced to date: From 30 conotoxin precursors and 6 gene superfamilies in *Conus (Textilia) bullatus* Linnaeus, 1758 [14], to 401 conotoxin precursors in *Conus (Harmoniconus) sponsalis* Hwass in Bruguière, 1792 [53] and 55 gene superfamilies in *Conus (Chelyconus) ermineus* Born, 1778 [54] (Table S3). Two studies reported even higher numbers of conotoxin precursors found in a single species: 662 in *Conus (Rhizoconus) miles* Linnaeus, 1758 [57] and 3305 in *Conus (Darioconus) episcopatus* da Motta, 1982 [58] (Table S3). These studies, reporting exceptionally high putative conotoxins numbers, may be the only ones that have been able to detect minor conotoxin variants already found in proteomic studies, but may also be a result of sequencing data processing [52,53]. This lack of homogeneity, along with the use of different bioinformatic pipelines and threshold criteria, the physiological intraspecific variation of the venom composition [11], and the lack of corroborating proteomic data for much of the reported findings, demands caution in comparing numbers resulting from different projects.

In conid literature, venom complexity has always been related to prey preference. However, recent studies [53,59] pointed out that conotoxin diversity is correlated to diet breadth more than prey type, revealing the tendency of species with more generalized diets to have more complex venoms and more predation-evoked venom genes. Almost nothing is known about *Profundiconus* ecology, but the limited data available suggest a worm-hunting diet with at least one species able to prey on fast moving molluscs [24,25]. The venom insulins found in *Profundiconus* are similar to those of worm- and mollusc-hunting *Conus* species and have an additional cysteine residue characteristic these species groups. As a result, based on transcriptomic analyses, *P.* cf. *vaubani* and *P. neocaledonicus* diets may include only a limited diversity of worm and/or mollusc preys.

### 3.3. Characterization of the First Putative Profunditoxin Gene Superfamilies Indicate They Are Divergent

Three completely new gene superfamilies were predicted, two of which are overexpressed in the VG, and therefore potentially represent main *Profundiconus* venom components. The high level of sequence divergence between the three gene superfamilies suggests three unrelated functions. Moreover, the presence of almost identical sequences of PFC-01 in both species may suggest a broader spread of this gene superfamily across *Profundiconus* species. Considering the phylogenetic position of *Profundiconus* as an independent lineage sister to the rest of the Conidae, these toxin superfamilies may be classified as “divergent” [60,61]. However, because of the high divergence of *Profundiconus* from the other cone snails, we expected to find more profunditoxins superfamilies, especially when considering that new *Conus* gene superfamilies continue to be routinely discovered, as recently reported for *C. tribblei* and *C. lenevati* [56,62]. 

### 3.4. Turripeptides Retained in Profundiconus Venom

In *Profundiconus*, turripeptide-related transcripts were among the most diversified neuropeptide classes. Turripeptides and conotoxins are thought to have a common evolutionary origin as they show similar precursor organization of signal, pro mature, and post regions [35]. However, when the first turrid venom peptides were discovered, little overlap was found with conotoxins, and the large majority of them belonged to new gene superfamilies, not yet found in conids [31,32,34,35]. Later, the discovery of peptides similar to conotoxins or turripeptides in distant taxa, likely as the results of convergent evolution, underlined the broader benefits obtained by recruiting them as venom or secretion components. For example, turripeptide-like toxins were found among the feeding-related proteins of the vampire snail *Colubraria reticulata* (Blainville, 1829) [63], in the hunting venom of bloodworms [64], and in the defensive one of fireworms [65], while conotoxin-like peptides were found in the mussel *Mytilus galloprovincialis* Lamarck, 1819 [66]. If the present distribution of turripeptides among taxa is considered in the context of the conoidean phylogeny, they might be present in the Conoidea common ancestor, and perhaps even earlier. Eventually, in the Conidae, some turripeptides (what we currently call conotoxins, found in both turrids and conids) started to rapidly diversify, becoming the prevalent component of conid venom, up to the complete loss of turripeptides at least in the genus *Conus*. The early divergence of *Profundiconus* from the rest of Conidae may explain why some turripeptide gene superfamilies are still retained in this genus. However, this hypothesis needs to be corroborated by characterizing the VG components of more *Profundiconus* species, and by a more extensive comparison of turripeptides from other groups belonging to the conid radiation, such as *Conasprella*, *Lilliconus*, and *Pygmaeoconus*, for which limited, or no data, are available.

## 4. Conclusions

This is the first study to explore the toxin diversity in the venom gland of the genus *Profundiconus*, the earliest offshoot of cone snails. In the *Profundiconus* species examined, *P.* cf. *vaubani* and *P. neocaledonicus*, putative conotoxin numbers were not particularly high, however the large majority of them belonged to potentially new gene superfamilies, with three gene superfamilies highly divergent from conotoxin superfamilies described to date. The presence of some rare conid gene superfamilies and the absence of those largely widespread in other cone snails, along with the occurrence of putative turripeptides and a relevant percentage of non-neuropeptides components, constitute uncharacteristic features of *Profundiconus* venom composition with respect to the other cones investigated to date. Importantly, the observation of prohormone-4 and lectins provide a first overview about *Profundiconus* venom being potentially involved in prey behavioural alteration and haemostasis impairing. Additionally, the low conotoxin diversity and the presence of a signalling-like venom insulin in all specimens offer clues about the poorly known trophic ecology of these species, suggesting that *P.* cf. *vaubani* and *P. neocaledonicus* may prey on a limited number of species, possibly worms or other molluscs. Finally, our results indicate that, even if several cone snail venom gland transcriptomes have been studied, we are still far from reaching the plateau of conotoxins diversity. The venom composition of *Profundiconus* calls for further investigation of venom gland transcriptomes in neglected Conidae lineages, and for a more detailed analysis of those from previously studied species, in order to fully understand the venom arsenal of this family and its ecological and evolutionary importance.

## 5. Materials and Methods

### 5.1. Sample Collection and Identification

Three specimens of the genus *Profundiconus* were collected during the expedition KANACONO (doi 10.17600/16003900; expeditions.mnhn.fr) in New Caledonia, South of the Isle of Pines: MNHN-IM-2013-66002 (st. DW4666, 22°53′ S, 167°16′ E, 530–545 m), MNHN-IM-2013-69343 and MNHN-IM-2013-69344 (st. DW4722, 22°54′ S, 167°16′ E, 496–505 m). The specimen MNHN-IM-2013-66343 was morphologically identified as *Profundiconus neocaledonicus* (*Pneo*) Tenorio & Castelin, 2016. This identification was then confirmed by sequencing the Barcode fragment of the COI gene (results not shown). The specimens MNHN-IM-2013-66002 and MNHN-IM-2013-66344 most probably correspond to a white form of *Profundiconus vaubani* (Röckel & Moolenbeek, 1995), and their COI sequences also match COI sequences of *P. vaubani* from New Caledonia (Tenorio & Castelin, 2016). We will then refer to them as *P*. cf. *vaubani* (*Pvau*). In this work, the three specimens are referred as following: *Pvau1* for MNHN-IM-2013-66002, *Pvau2* for MNHN-IM-2013-69344, and *Pneo* for MNHN-IM-2013-69343. COI sequences were deposited in GenBank (accession numbers MN540394-6).

### 5.2. RNA Extraction, Sequencing, and De Novo Assembly

For *Pvau2* and *Pneo*, a piece of foot was isolated, and for all the three specimens, the venom gland was dissected after crushing the shells (the shell remains were kept as vouchers). RNA was extracted using the trizol method. Bioanalyzer traces were used to assess total RNA quality and determine suitability for sequencing. The cDNA libraries were prepared and sequenced at the New York Genome Center. Libraries were prepared using the automated polyA RNAseq library prep protocol and sequenced with Illumina HiSeq 4000 with 150-bp paired-end reads. Trimmomatic v.0.36 [67] was used to remove adapter contamination and filter low-quality reads (ILLUMINACLIP option enabled, seed mismatch threshold = 2, palindrome clip threshold = 40, simple clip threshold of 15; SLIDING WINDOW option enabled, window size = 4, quality threshold = 20; MINLEN = 36; LEADING = 3; TRAILING = 3). Reads were merged using FLASH v.1.2.8 [68] with a min overlap parameter of 5, a max overlap parameter of 100, and a mismatch ratio of 0.05. Trinity v.2.4 was then used to assemble transcripts [69]. CAP3 [70] was finally used with default parameters and cd-hit v.4.6 (percent identity = 99%; [71]) to reduce redundancy in the assemblies. Transcriptome data are available at GenBank (BioProject PRJNA574281).

### 5.3. Transcriptome Annotation and Differential Expression Analysis

To identify *Profundiconus* venom components, a slightly modified standard pipeline was used [65,72]. Briefly, VG contigs were searched for all possible open reading frames (ORFs) with a minimum length of 10 aa using a homemade script (Script 1), and signal sequences were identified using SignalP. Two similarity searches (e-value 10^−5^) were then performed in parallel on filtered VG transcripts. The first was made using BLASTp v.2.6 (National Center for Biotechnology Information, Bethesda, MD, USA) [73] against a customized toxin database, built using proteins from ConoServer [60,74], UniProt Animal Toxin Annotation Project [75], and teretoxin sequences from Gorson et al. [72]. For the second, transcripts were searched against hidden Markov models (HMM) profiles built on toxins from the protein family Pfam database [76], using HMMER v.3.2 [77]. Pfam database was chosen as a complement to the homemade database because, being built through a different search strategy that takes into consideration the full protein domain similarity (e.g., also less-conserved regions and insertions), it includes in the toxin category sequences belonging to a wider range of organisms. This approach is particularly important for finding putative venom components in a genus, like *Profundiconus*, never studied before.

Transcript per million (TPM) values were calculated using Kallisto [78] for all the VG and F contigs of the two specimens for which both tissues were sequenced (*Pvau2* and *Pneo*) (bootstrap = 100). Differential expression analyses with technical replicates were then performed between the contigs of the VG and the F of the same specimen, using the R package NOISeq v.2.22 (NOISeq-sim function, normalization = rpkm, length correction = 1 [79]). Those contigs overexpressed in the VG were added to the dataset, if not already present, after ORFs and signal sequences searches. All selected transcripts were searched using BLASTp against UniProt full database and NCBI nr database. Results were manually inspected, and transcripts with a higher hit to a non-toxin protein family were discarded. DeepLoc v.1 (protein encoding = Profiles [80]) was used to predict the subcellular localization of the remaining contigs, and only the extracellular ones were retained. Finally, identical contigs (100%) were identified with cd-hit v.4.6 [71], and duplicates within a single transcriptome were discarded. Protein sequences were deposited in UniProt.

OrthoVenn2 web platform [81] was used to compare the compounds expressed in the VG and F of *Pvau2* and *Pneo* by grouping the ORF obtained into putative orthologues clusters (e-value 10^−5^) and annotating them using Gene Ontology biological classes.

### 5.4. Venom Component Identification and Diversity

Particular attention was given to proteins similar to previously identified venom components and to those overexpressed in the venom glands. When possible, for each transcript, the hypothetical molecular type, the protein family, and a potential molecular target were identified through sequence (BLASTp and HMMER) and structural (SMART [82] and SWISS-MODEL [83]) comparison with already-described proteins. All these assignations have to be considered preliminary, until confirmed by proteomics and bioactivity tests; consequently, all the functional annotations we propose have also to be considered putative. VG transcripts were divided into three putative functional groups: Components interfering with the physiology of the prey (toxins), those in charge of toxin post-translation modifications and folding, and peptides facilitating toxin spread in prey’s body. The toxin group included bioactive compounds putatively assigned to several molecular types: Neuropeptides, hydrolases, lectins, oxidoreductases, and protease inhibitors. Among them, the venom neuropeptide category included predicted peptides hypothetically affecting prey’ nervous system (conotoxins and turripeptides), muscular system (cardioactive peptides), immune system (allergens), or causing hypoglycaemic shock (insulins). The rest of the toxins were instead targeting prey’ hemostasis system (e.g., kunitz-type serine protease inhibitors, multicopper oxidases, C-type lectins, and trypsin-like serine proteases). The other two venom component categories respectively included oxidoreductases involved in toxin post-translation modifications and folding (e.g., pyridine nucleotide-disulfide oxidoreductase), and hydrolases easing toxins spreading (e.g., chitinase, astacins, and neprilysins). *Profundiconus* venom components were considered new when including at least one different aa compared to known ones.

Those *Profundiconus* transcripts similar to conotoxins and turripeptides, principal components of other conoidean venoms, were further investigated for identifying gene superfamilies and cysteine patterns. Conotoxin preliminary gene superfamily designations were based on results obtained from BLASTp and HMMER. The full sequences were analyzed with BLASTp, while the signal sequences only were searched against profiles of conotoxin precursor signal sequences retrieved from ConoServer. Gene superfamily hypothetical assignation, or both results in case of discordance between the two methods used, were tested in the following step. The average percentage sequence identity (=similarity score) of the signal region was calculated between each transcript and all conotoxins ascribed to the same preliminary gene superfamily (pPID). For each gene superfamily, the average percentage sequence identity of the signal sequences (sPID) and its standard deviation (sSD) were also calculated. A transcript was considered belonging to a known gene superfamily when pPID > sPID - sSD. For some small gene superfamilies, exceptionally high sPID values were retrieved. However, these values can be an underestimation of the real gene superfamilies diversity, mainly due to the fact that only a few sequences are available and for a limited number of *Conus* species. For this reason, in those gene superfamilies with a sPID > 76% (the average sPID value of the biggest conotoxin superfamily [9]), this score was instead used as a threshold. All transcripts showing similarity to conotoxins but with a pPID score under the respective threshold were assigned to the gene superfamily “X-like”, where X represented the most similar gene superfamily. Sequences showing an exceptional low pPID value (<40% as used in ConoServer) were instead categorized in completely new gene superfamilies (“PFC-XX”, for ProFundiConus-XX), and in this work named “profunditoxins” (defined as natural peptide toxins produced by *Profundiconus*, by analogy with conotoxins, turritoxins, and teretoxins [84,85]). Conotoxins and profunditoxins diversity was measured by Shannon’s diversity index (H’) and homogeneity among gene superfamilies were calculated by Shannon’s evenness index (E) (as in [62]) using Past3 [86].

For turripeptides, a unique and broadly used gene superfamily classification scheme is not available. Consequently, we calculated the percentage of sequence identity for the signal region between each transcript resulting similar to a turripeptide with BLASTp or HMMER and known turripeptides (tPID). Those transcripts with tPID > 76% were labelled as “turripeptide”, and the rest as “turripeptide-like”. A homemade script (Script 2) was used to identify cysteine pattern of conotoxins, conotoxin-like, profunditoxins, turripeptides, and turripeptide-like mature region. For the other protein families found in *Profundiconus*, homologous sequences from other molluscs and venomous animals were retrieved from NCBI and UniProt and the domain region was aligned using MAFFT v.7 (E-INS-i algorithm [87,88]). For insulin, *Profundiconus* chain A and B were aligned with those from 17 *Conus* species grouped by diet (fish, mollusc, and worm) (Figure S5). Intra and inter groups identity and similarity (Blosum62) percentages were then calculated.

## Figures and Tables

**Table 1:** Cysteine frameworks found in conotoxin, profunditoxin, and turripeptide gene superfamilies. ^1^ indicates cysteine frameworks never described before for that gene superfamily. Numbers represent total transcripts found in each VG for each gene superfamily, and between parentheses those overexpressed in the VG (if any) for the two specimens for which differential expression analysis were conducted (*Pvau2* and *Pneo*).

Gene Superfamily	Pvau1	Pvau2	Pneo	Cysteine Framework
A-like	1	2 (1)	2 (1)	VI/VII, IX ^1^
B1-like	1	0	2 (1)	0
C-like	1	2 (1)	2 (1)	0
G2-like	0	1	1	VI/VII ^1^
H	1	1 (1)	2 (1)	VIII ^1^, C-C ^1^
I1	0	0	1 (1)	XI
I1-like	0	1	3 (1)	XI, 0 ^1^
I2	2	2	1 (1)	VI/VII^1^, XI, C-C-C-CC-CC ^1^, C-C-C-CC-CC-C ^1^
I2-like	0	0	1	XI
I3-like	1	1 (1)	2 (1)	XI, XV ^1^
L	0	2 (1)	0	XIV
L-like	1	0	0	XIV
M	4	5	7	0, VI/VII, XIII ^1^, XXII ^1^, C-C ^1^, C-C-C ^1^, C-CC-C-CC-C-C ^1^
M-like	1	3	1	0, VI/VII, XV ^1^
O1	5	6 (2)	2 (2)	0 ^1^, VI/VII, XVI
O2-like	2	3 (2)	1 (1)	VI/VII, XV
O3-like	5	8 (2)	17 (6)	0, VI/VII, XI ^1^, XIV ^1^, C^1^, C-C-C-C-C ^1^
P	4	5 (2)	2 (1)	0 ^1^, XXV ^1^, IX ^1^, C-C-C-C-C ^1^
P-like	0	1	0	IX
Q-like	0	0	1 (1)	XVI
T	0	2	0	0 ^1^
Y-like	3	3 (1)	2	VI/VII ^1^
Div.M---L-LTVA	2	2 (1)	3 (1)	VI/VII, IX
Div.MSTLGMTLL-	1	1	1 (1)	XV ^1^, C-C-CC-CC-C-C-C ^1^
PFC-01	1	1	1 (1)	XIV
PFC-02	0	1	0	VI/VII
PFC-03	1	1 (1)	0	VIII
Turripeptide	3	6	4	0, XVI, IX, C
Turripeptide-like	0	1	3	IX

**Table 2:** Comparison of *Profundiconus* A and B insulin chains. *Profundiconus* species are compared to *Conus* species grouped by diet (fish-, mollusc-, or worm-hunters), and maximum and minimum values are reported. Identity and similarity percentage inside each diet group are also listed (Intragroup). In bold, the highest identity/similarity values for each combination and, indicated by a number, the *Conus* species to which they refer: ^1-^*Conus (Cylinder) textile* Linnaeus, 1758, ^2-^*Conus (Cylinder) victoriae* Reeve, 1843, ^3-^*Conus (Strategoconus) varius* Linnaeus, 1758, ^4-^*Conus (Lividoconus) floridulus* A. Adams & Reeve, 1848, ^5-^*Conus (Virroconus) ebraeus* Linnaeus, 1758, ^6-^*Conus (Strategoconus) planorbis* Born, 1778, and ^7-^*Conus (Virgiconus) virgo* Linnaeus, 1758).

		Intragroup		*Pneo*		*Pvau*	
Insulin	*Conus* prey	% Id.	% Sim.	% Id.	% Sim.	% Id.	% Sim.
Chain A	Fish	43–95	62–100	38–43	52–67	26–32	52–64
	Mollusc	81–100	90–100	68–81 ^1^	76–90 ^1,2^	41–50	65–76
	Worm	44–100	72–100	52–76	68–86	38–56 ^5^	71–85 ^4,6^
Chain B	Fish	59–100	91–100	23–22	48–64	38–43	52–67
	Mollusc	50–97	79–97	41–50 ^1^	62–74	69–81 ^1^	88–90
	Worm	32–100	53–100	41–50 ^3,4^	62–82 ^4^	52–77	81–92 ^7^

## Supplementary Materials

The following are available online at https://www.mdpi.com/2072-6651/11/11/623/s1, Figure S1: Graphical summary of clusters obtained for each sample, Figure S2: Pie-charts of transcripts putative molecular types and effect/target for each specimen, Figure S3: Plots of differential expression results between venom gland and foot of *Pneo* and *Pvau2*, Figure S4: Venn diagram of conotoxins and profunditoxins found in the VG of *Profundiconus* specimens, Figure S5: Alignment of insulin chain A and B, Figure S6: Serine protease domain alignment of sequence from *Profundiconus*, bivalves and snakes, Table S1: Sequencing, assembling and pipeline numbers for each tissue sample analyzed, Table S2: List of putative venom component found in the VG of *Pvau1*, *Pvau2*, and *Pneo* with molecular identification and amino acidic sequences, Table S3: Comparison of conotoxins, profunditoxins and gene superfamilies found in *P. neocaledonicus* and *P.* cf. *vaubani* with those of other conids, Script 1: Python script for identifying ORF in transcript sequences, Script 2: Python script for identifying cysteine patterns in amino acid sequences.
