# Supplementary material for: Venom Diversity and Evolution in the Most Divergent Cone Snail Genus Profundiconus
Source: Toxins (Basel). 2019 Oct 28;11(11):623. doi: 10.3390/toxins11110623 (PMC6891753; doi:10.3390/toxins11110623)
Supplement: Supplementary file 1 [file toxins-11-00623-s001.zip › toxins-623515 supplementary/toxins-623515 supplementary proofdone.docx]

Supplementary Materials: Venom Diversity and Evolution in the Most Divergent Cone Snail Genus *Profundiconus*

Giulia Fassio, Maria Vittoria Modica, Lou Mary, Paul Zaharias, Alexander E. Fedosov, Juliette Gorson, Yuri I. Kantor, Mandё Holford and Nicolas Puillandre

**Table S1.** Sequencing, assembling and pipeline numbers for each tissue sample analysed.

| Voucher | Species | Sample | Tissue | Raw Reads | Contigs | ORFs | SignalP | Putative Venom Components |
| --- | --- | --- | --- | --- | --- | --- | --- | --- |
| MNHN-IM-2013-66002 | *P*. cf. *vaubani* | Pvau1 | VG | 32,318,063 | 101,363 | 2,009,743 | 38,745 | 51 |
| MNHN-IM-2013-69344 | *P*. cf. *vaubani* | Pvau2 | VG | 32,496,724 | 172,705 | 5,237,063 | 87,390 | 108 |
| MNHN-IM-2013-69343 | *P. neocaledonicus* | Pneo | VG | 39,048,347 | 127,617 | 3,824,173 | 65,072 | 86 |
| MNHN-IM-2013-69344 | *P*. cf. *vaubani* | Pvau2 | F | 36,230,698 | 267,698 | 9,301,844 | - | - |
| MNHN-IM-2013-69343 | *P. neocaledonicus* | Pneo | F | 39,488,251 | 380,364 | 11,747,777 | - | - |

**Table S3.** Comparison of conotoxins, profunditoxins and gene superfamilies found in P. neocaledonicus and P. cf. vaubani (in bold) with those of other conids. ^1^ indicates species sequenced in more than one article. VG = venom gland, VB = venom bulb, SG = salivary gland, RS = radular sac.

| **Species** | **Conotoxin** | **Gene Superfamilies** | **Samples sequenced** | **Pool (If Applicable)** | **Body Part Sequenced** | **Sequencing Platform** | **Reference** |
| --- | --- | --- | --- | --- | --- | --- | --- |
| *Conus (Textilia) bullatus* | 30 | 6 | several | 1 | VG | Roche 454 / Illumina HiSeq2000 | [1] |
| *Conus (Pionoconus) consors* | 53 | 11 | several | 1 | VG | Roche 454 | [2] |
| *Profundiconus neocaledonicus* | 55 | 21 | 1 |  | VG | Illumina HiSeq 2000 | **Present study** |
| *Conus (Gastridium) geographus^1^* | 63 | 16 | 4 | 4, by VG segments | VG | Roche 454 | [3] |
| *Conus (Stephanoconus) imperialis* | 70 | 20 | 1 |  | VG | Illumina HiSeq 2000 | [4] |
| *Conus (Gastridium) geographus^1^* | 75 | 21 | 1 |  | VG | Illumina HiSeq 2000 | [5] |
| *Profundiconus* cf. *vaubani* | 75 | 24 | 2 |  | VG | Illumina HiSeq 2000 | **Present study** |
| *Conus (Virroconus) ebraeus* | 75 | 27 | 1 |  | VG | Illumina HiSeq 2000 | [4] |
| *Conus (Conus) marmoreus^1^* | 81 | 14 | 1 |  | VG | Illumina HiSeq 2000 | [4] |
| *Conus (Puncticulis) pulicarius* | 82 | 14 | 1 |  | VG | Roche 454 | [6] |
| *Conus (Lividoconus) quercinus^1^* | 97 | 25 | 1 |  | VG | Illumina HiSeq 2000 | [4] |
| *Conus (Splinoconus) tribblei^1^* | 100 | 39 | 3 |  | VG | Illumina HiSeq 2000 | [7] |
| *Conus (Rhizoconus) rattus* | 102 | 28 | 1 |  | VG | Illumina HiSeq 2000 | [4] |
| *Conus (Cylinder) gloriamaris* | 108 | 31 | 1 |  | VG | Illumina HiSeq 2000 | [8] |
| *Conus (Cylinder) victoriae* | 113 | 20 | several | 1 | VG | Roche 454 | [9] |
| *Conus (Virgiconus) virgo* | 113 | 25 | 1 |  | VG | Illumina HiSeq 2000 | [4] |
| *Conus (Splinoconus) lenavati* | 132 | 40 | 3 |  | VG | Illumina HiSeq 2000 | [7] |
| *Conus (Lividoconus) quercinus^1^* | 133 | 34 | 3 | 3, by body parts | VG, VB, SG | Illumina HiSeq 4000 | [10] |
| *Conus (Splinoconus) tribblei^1^* | 136 | 36 | 20 | 1 | VG | Roche 454 / Illumina HiSeq2000 | [11] |
| *Conus (Turriconus) andremenezi* | 196 | 25 | 2 |  | VG | Illumina HiSeq 2000 | [5] |
| *Conus (Strategoconus) varius* | 198 | 29 | 1 |  | VG | Illumina HiSeq 2000 | [4] |
| *Conus (Dendroconus) betulinus* | 215 | 37 | 8 | 6, by body parts and snail size | VG | Sanger ABI 3730 / Illumina HiSeq2000 | [12] |
| *Conus (Lividoconus) lividus* | 244 | 31 | 1 |  | VG | Illumina HiSeq 2000 | [4] |
| *Conus (Turriconus) praecellens* | 250 | 29 | 2 |  | VG | Illumina HiSeq 2000 | [5] |
| *Conus (Conus) marmoreus^1^* | 264 | 26 | 1 |  | VG | Roche 454 | [13,14] |
| *Conus (Puncticulis) arenatus* | 326 | 36 | 1 |  | VG | Illumina HiSeq 2000 | [4] |
| *Conus (Virroconus) coronatus* | 331 | 32 | 1 |  | VG | Illumina HiSeq 2000 | [4] |
| *Conus (Chelyconus) ermineus* | 378 | 55 | 3 | 3, by VG segments | VG | Illumina HiSeq 2500 | [15] |
| *Conus (Harmoniconus) sponsalis* | 401 | 35 | 1 |  | VG | Illumina HiSeq 2000 | [4] |
| *Conus (Rhizoconus) miles* | 662 | 16 | 1 |  | VG | Roche 454 | [16] |
| *Conus (Gastridium) tulipa* | 764 | 18 | 2 |  | VG | Roche 454 | [17] |
| *Conus (Darioconus) episcopatus* | 3305 | 25 | 1 |  | VG, RS, SG | Illuminq MiSeq | [18] |


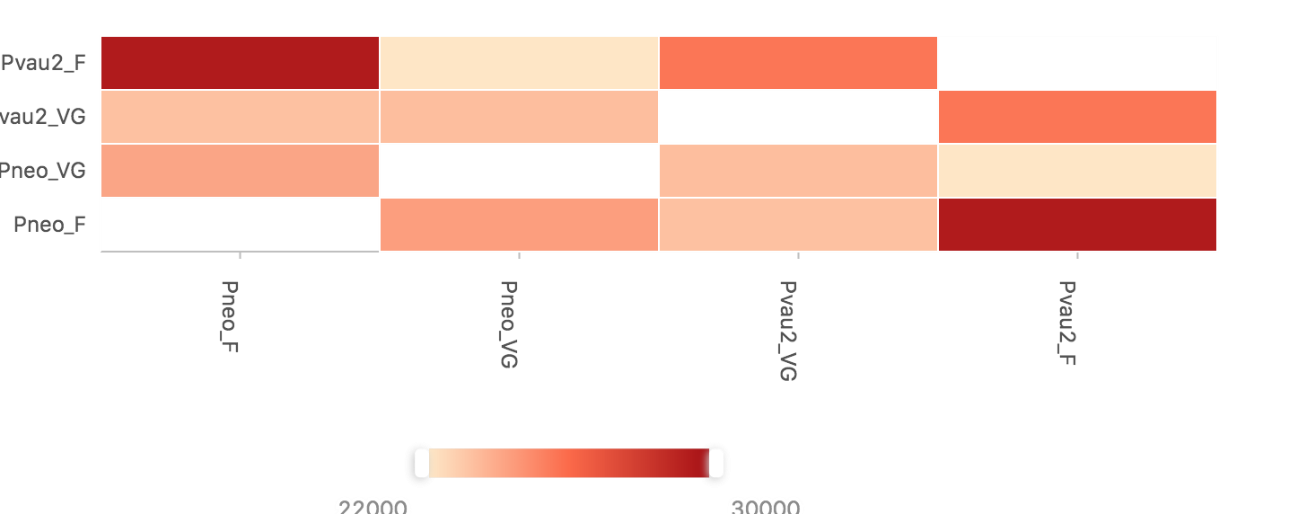

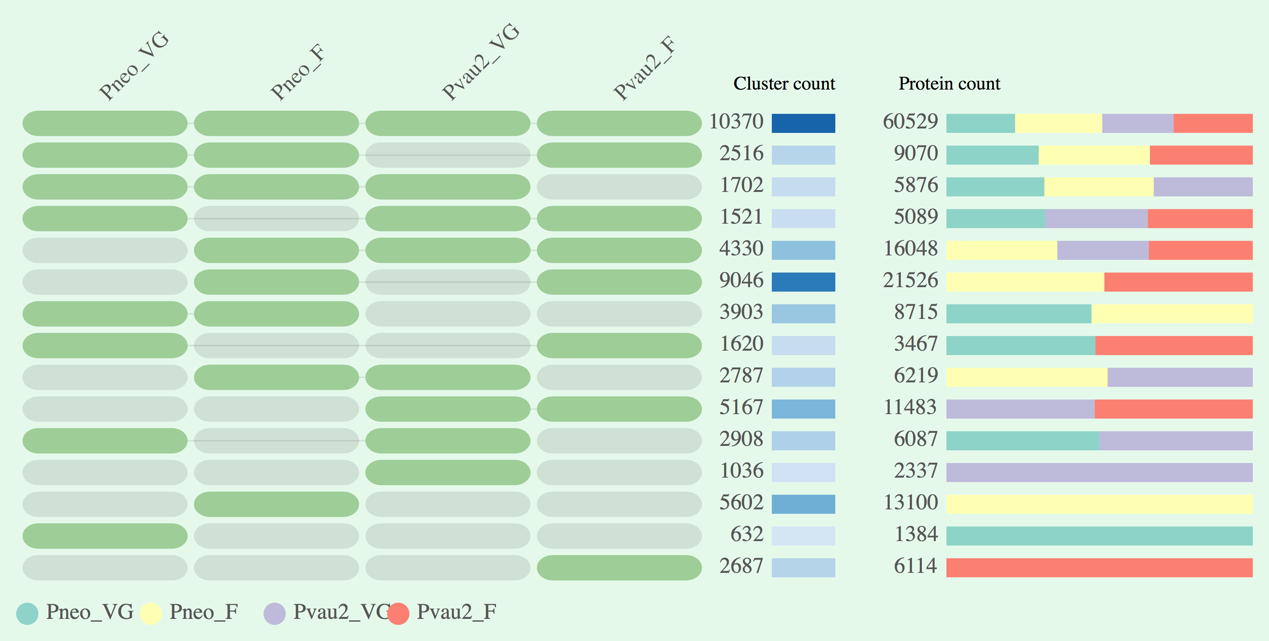

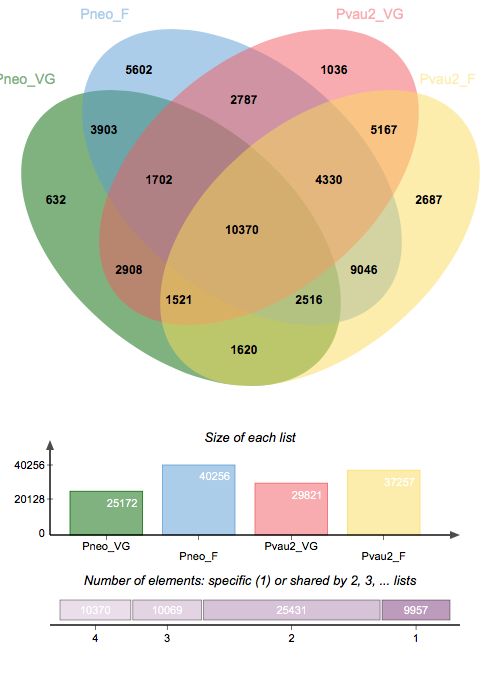


**Figure S1.** Graphical summaries of clusters obtained for each sample by OrthoVenn2. Top-down: 1) heatmap of clusters shared between samples; 2) count of clusters for each sample; 3) Venn diagram of clusters.

**Figure S2.** Pie-charts of transcripts putative molecular types (first raw) and effect/target (second raw) for each specimen.


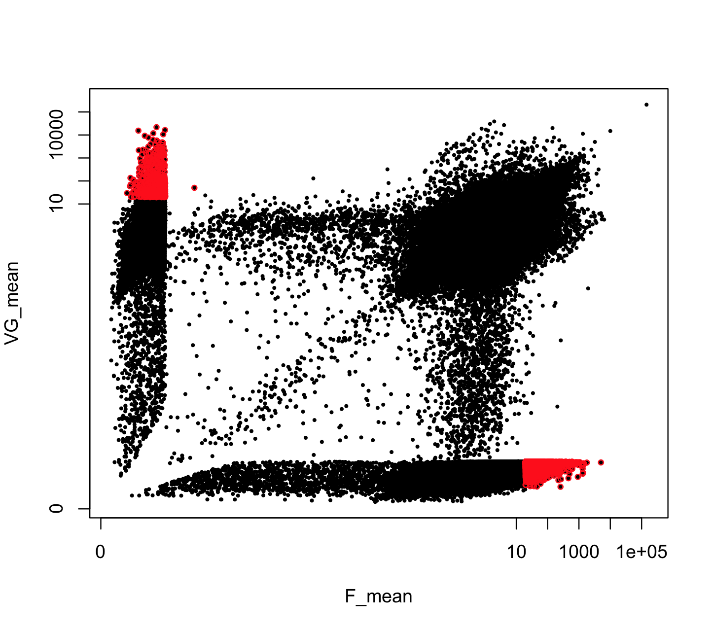

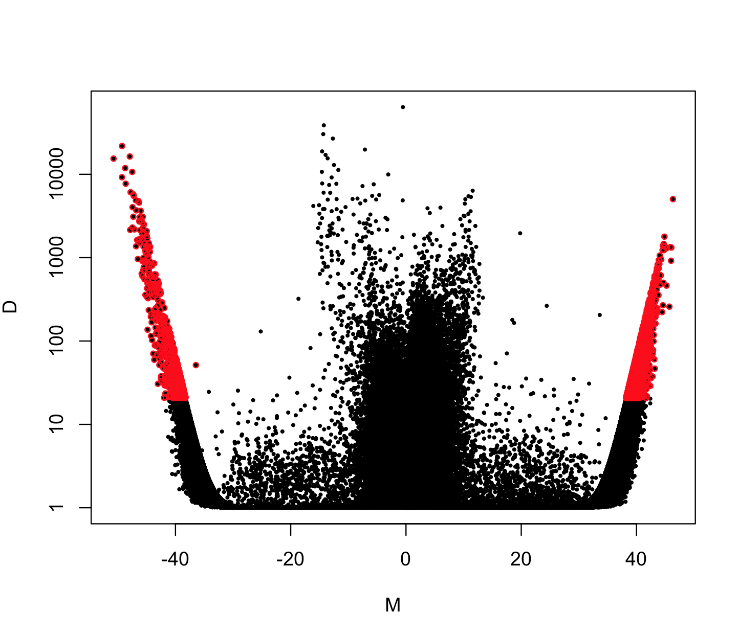

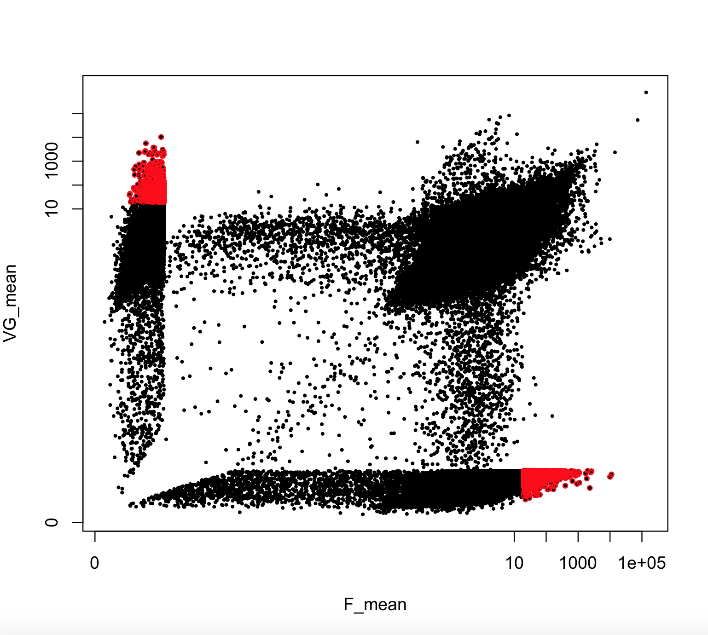

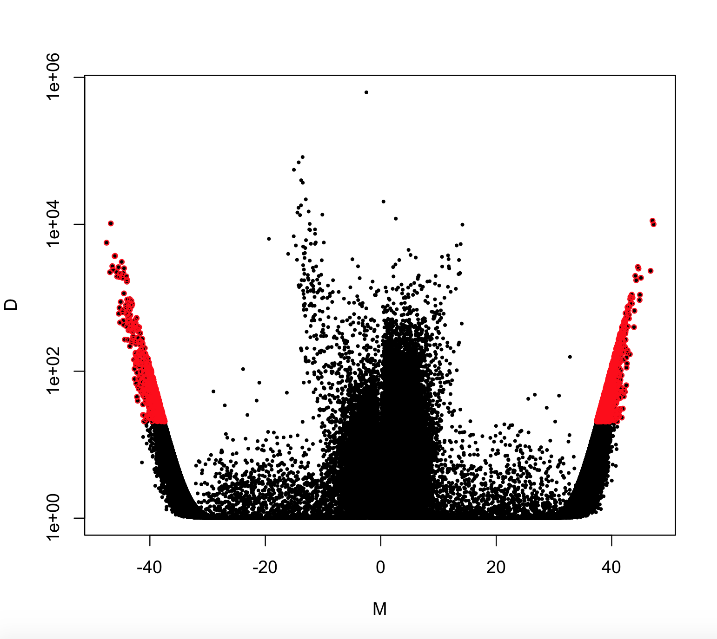


**Figure S3.** Plots of differential expression results between venom gland (VG) and foot (F) of Pneo (first raw) and Pvau2 (second raw). First column: expression plots showing average expression values of each contig in the F (x-axis) and VG (y-axis). Second column: MD plot showing log2-fold change (M = the base 2 logarithm of the ratio between a contig expression value in the F and in the VG; x-axis) and the difference in expression (log2-scale of absolute value) between F and VG (D; y-axis) for each contig. Differentially expressed contigs are highlighted in red.


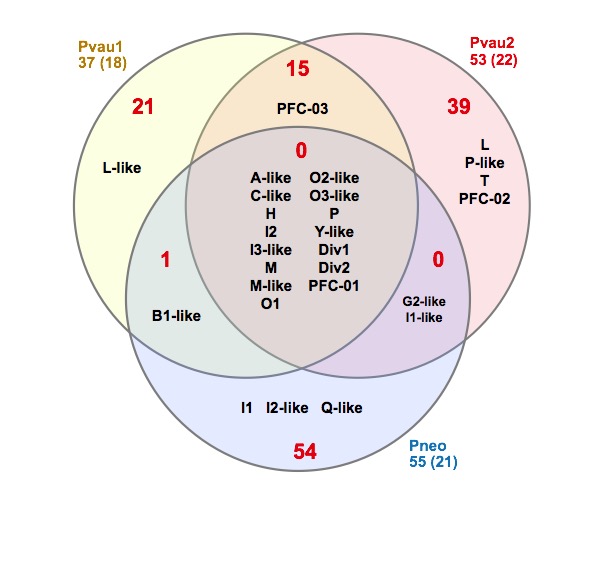


**Figure S4.** Venn diagram of conotoxins and profunditoxins found in the VG of Profundiconus specimens. In each set, numbers indicate the total count of unique conotoxins while names indicate gene superfamilies. For each specimen, the total number of conotoxins and total number of gene superfamilies (a number in parentheses) are indicated.

**Figure S5.** MAFFT alignment of insulin chain A (above) and chain B (below) from *Profundiconus* and 17 *Conus* species. Letters at the end of the names indicates species diets: F = fish, S = snails, and W = worms. Amino acids are coloured in grey scale based on Blosum62 similarity score (black 100%).


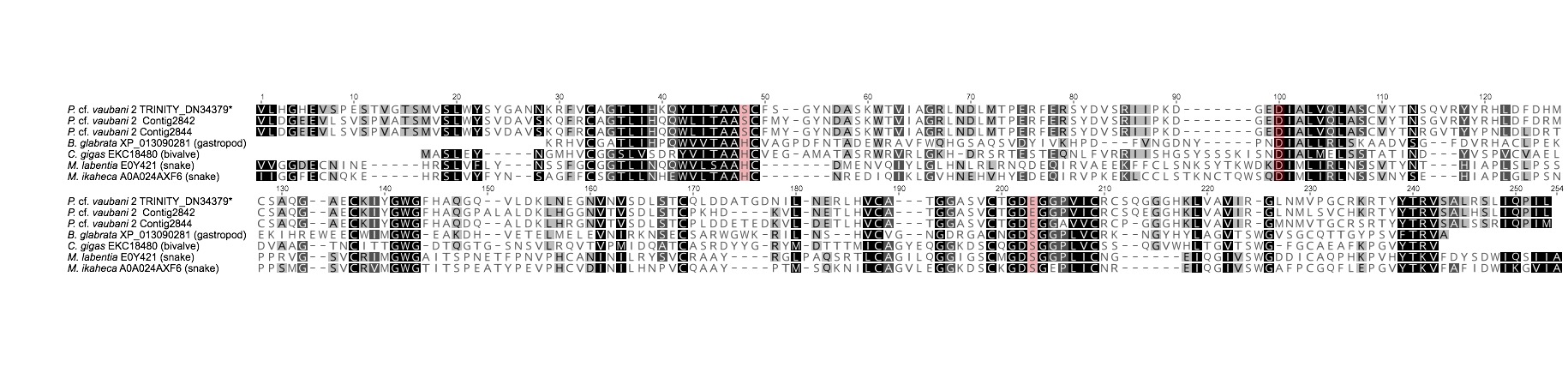


**Figure S6.** Serine protease domain alignment of sequence from Profundiconus, bivalves and snakes. Highlighted in red the conserved catalytic triad. Amino acids are coloured in grey scale based on Blosum62 similarity score (black 100%). Asterisk indicates the Profundiconus sequence overexpressed in the venom gland.

**Script 1.** Python script for identifying ORF in transcript sequences.

#!/usr/bin/python3

####################################################

## usage : python extract_orfs.py sequences.fasta ##

########## requiert : python3 ; biopython ##########

####################################################

import Bio

import sys

from Bio import SeqIO

meth=['ATG']

stop=['TAA','TAG','TGA']

with open(sys.argv[1],"rU") as handle:

with open("orfs.fasta","w") as outfile:

for record in SeqIO.parse(handle,"fasta"):

seq=record.seq

table=1

min_pro_len=10 #taille minimum des orfs (en AA)

for strand, nuc in [(+1, seq), (-1, seq.reverse_complement())]:

for frame in range(3):

i=0

b1=str(nuc[frame:])

#print("{}\n".format(b1))

b2=(nuc[frame:]).translate(table).split("\n")

#print("{}\n".format(b2[0]))

debut=0

while i<len(b1):

cds=""

if b1[i:i+3] in stop:

pos_stop=i

cds=(nuc[frame:])[debut:].translate(table, to_stop=True).split("*")

if len(cds[0])>=min_pro_len:

outfile.write(">{}_strand{}_frame{}_coord{}-{}\n".format(record.id,strand,frame+1,debut,pos_stop))

outfile.write("{}\n".format(str(cds[0])))

debut=i+3

i+=3

if cds=="":

if (i-debut)>=min_pro_len*3:

cds=(nuc[frame:])[debut:].translate(table).split("\n")

outfile.write(">{}_strand{}_frame{}_coord{}-{}\n".format(record.id,strand,frame+1,debut,i))

outfile.write("{}\n".format(str(cds[0])))

**Script 2.** Python script for identifying cysteine patterns in amino acid sequences.

#! /usr/bin python3

# coding: utf-8

from Bio import SeqIO

import re

import os

import sys

dico={}

dico2={}

liste_patterns_cys={"C-CC-C-C-C":"XXVII","CC-C-C":"I","CCC-C-C-C":"II","CC-C-C-CC":"III","CC-C-C-C-C":"IV","CC-CC":"V","C-C-CC-C-C":"VI_VII","C-C-C-C-C-C-C-C-C-C":"VIII","C-C-C-C-C-C":"IX","C-C-CC-CC-C-C":"XI","C-C-C-C-CC-C-C":"XII","C-C-C-CC-C-C-C":"XIII","C-C-C-C":"XIV","C-C-CC-C-C-C-C":"XV","C-C-CC":"XVI","C-C-CC-C-CC-C":"XVII","C-C-CC-CC":"XVIII","C-C-C-CCC-C-C-C-C":"XIX","C-CC-C-CC-C-C-C-C":"XX","C-C-C-C-C-C-C-C":"XXII","C-C-C-CC-C":"XXIII","C-CC-C":"XXIV","C-C-C-C-CC":"XXV","C-C-C-C-CC-CC":"XXVI"}

with open(sys.argv[1],'r') as infile:

for record in SeqIO.parse(infile,"fasta"):

dico[record.id]=str(record.seq)

for key,value in dico.items():

test=re.sub("[-]$","",(re.sub("^[-]", "", (re.sub("[-]+", "-", (re.sub("[^C]", "-", value)))))))

if test in liste_patterns_cys.keys():

dico2[key]=liste_patterns_cys[test]

else:

dico2[key]=test

with open(sys.argv[2],'w') as outfile:

outfile.write("ID\tSequence\tPattern\n")

for key,value in dico2.items():

outfile.write("{}\t{}\t{}\n".format(key,dico[key],dico2[key]))

References

1. Hu, H.; Bandyopadhyay, P.K.; Olivera, B.M.; Yandell, M. Characterization of the Conus bullatus genome and its venom-duct transcriptome. *BMC Genomics* **2011**, *12*, 60–75.
2. Terrat, Y.; Biass, D.; Dutertre, S.; Favreau, P.; Remm, M.; Stöcklin, R.; Piquemal, D.; Ducancel, F. Toxicon High-resolution picture of a venom gland transcriptome : Case study with the marine snail Conus consors. *Toxicon* **2012**, *59*, 34–46.
3. Hu, H.; Bandyopadhyay, P.K.; Olivera, B.M.; Yandell, M. Elucidation of the molecular envenomation strategy of the cone snail Conus geographus through transcriptome sequencing of its venom duct. *BMC Genomics* **2012**, *13*, 284.
4. Phuong, M.A.; Mahardika, G.N.; Alfaro, M.E. Dietary breadth is positively correlated with venom complexity in cone snails. *BMC Genomics* **2016**, *17*, 401.
5. Li, Q.; Barghi, N.; Lu, A.; Fedosov, A.E.; Bandyopadhyay, P.K.; Lluisma, A.O.; Concepcion, G.P.; Yandell, M.; Olivera, B.M.; Safavi-hemami, H. Divergence of the Venom Exogene Repertoire in Two Sister Species of Turriconus. *Genome Biol. Evol.* **2017**, *9*, 2211–2225.
6. Lluisma, A.O.; Milash, B.A.; Moore, B.; Olivera, B.M.; Bandyopadhyay, P.K. Novel venom peptides from the cone snail Conus pulicarius discovered through next-generation sequencing of its venom duct transcriptome. *Mar. Genomics* **2012**, *5*, 43–51.
7. Barghi, N.; Concepcion, G.P.; Olivera, B.M.; Lluisma, A.O. Comparison of the Venom Peptides and Their Expression in Closely Related Conus Species: Insights into Adaptive Post-speciation Evolution of Conus Exogenomes. *Genome Biol. Evol.* **2015**, *7*, 1797–1814.
8. Robinson, S.D.; Li, Q.; Bandyopadhyay, P.K.; Gajewiak, J.; Yandell, M.; Papenfuss, A.T.; Purcell, A.W.; Norton, R.S.; Safavi-hemami, H. General and Comparative Endocrinology Hormone-like peptides in the venoms of marine cone snails. *Gen. Comp. Endocrinol.* **2017**, *244*, 11–18.
9. Robinson, S.D.; Safavi-hemami, H.; Mcintosh, L.D.; Purcell, A.W.; Norton, R.S.; Papenfuss, A.T. Diversity of Conotoxin Gene Superfamilies in the Venomous Snail , Conus victoriae. *PLoS One* **2014**, *9*, e87648.
10. Gao, B.; Peng, C.; Zhu, Y.; Sun, Y.; Zhao, T.; Huang, Y.; Shi, Q. High Throughput Identification of Novel Conotoxins from the Vermivorous Oak Cone Snail ( Conus quercinus ) by Transcriptome Sequencing. *Int. J. Mol. Sci.* **2018**, *19*, 1–17.
11. Barghi, N.; Concepcion, G.P.; Olivera, B.M.; Lluisma, A.O. High Conopeptide Diversity in Conus tribblei Revealed Through Analysis of Venom Duct Transcriptome Using Two High-Throughput Sequencing Platforms. *Mar. Biotechnol.* **2015**, *17*, 81–98.
12. Peng, C.; Yao, G.; Gao, B.; Fan, C.; Bian, C.; Wang, J.; Cao, Y.; Wen, B.; Zhu, Y.; Ruan, Z.; et al. High-throughput identification of novel conotoxins from the Chinese tubular cone snail ( Conus betulinus ) by multi- transcriptome sequencing. *Gigascience* **2016**, 1–14.
13. Lavergne, V.; Dutertre, S.; Jin, A.; Lewis, R.J.; Taft, R.J.; Alewood, P.F. Systematic interrogation of the Conus marmoreus venom duct transcriptome with ConoSorter reveals 158 novel conotoxins and 13 new gene superfamilies. *BMC Genomics* **2013**, *14*, 780–792.
14. Dutertre, S.; Jin, A.; Kaas, Q.; Jones, A.; Alewood, P.F.; Lewis, R.J. Deep Venomics Reveals the Mechanism for Expanded Peptide Diversity in Cone Snail Venom. *Mol. Cell. Proteomics* **2013**, *12*, 312–329.
15. Abalde, S.; Tenorio, M.J.; Afonso, C.M.L.; Zardoya, R. Conotoxin Diversity in Chelyconus ermineus (Born, 1778) and the Convergent Origin of Piscivory in the Atlantic and Indo-Pacific Cones. *Genome Biol. Evol.* **2018**, *10*, 2643–2662.
16. Jin, A.; Kaas, Q.; Lavergne, V.; Kubala, P.; Lewis, R.J.; Alewood, P.F. Transcriptomic Messiness in the Venom Duct of Conus miles Contributes to Conotoxin Diversity. *Mol. Cell. Proteomics* **2013**, *12*, 3824–3833.
17. Dutt, M.; Dutertre, S.; Jin, A.-H.; Lavergne, V.; Alewood, P.F.; Lewis, R.J. Venomics Reveals Venom Complexity of the Piscivorous Cone Snail, Conus tulipa. *Mar. Drugs* **2019**, *17*, 1–18.
18. Lavergne, V.; Harliwong, I.; Jones, A.; Miller, D.; Taft, R.J.; Alewood, P.F. Optimized deep-targeted proteotranscriptomic profiling reveals unexplored Conus toxin diversity and novel cysteine frameworks. *Proc. Natl. Acad. Sci.* **2015**, *112*, E3782–E3791.
